# The incidence of bone marrow oedema at the sacroiliac joints in a non-rheumatological population — a retrospective cohort study

**DOI:** 10.1186/s12891-019-2978-1

**Published:** 2019-12-07

**Authors:** A. Nygaard, A. G. Jurik, C. Lund, B. Schiøttz-Christensen

**Affiliations:** 1 Spine Centre of Southern Denmark, Research Department, Lillebaelt Hospital, Oestre Hougvej 55, 5500 Middelfart, Denmark; 2 Institute of Regional Health Research, University of Southern Denmark, Odense, Denmark; 3 Department of Radiology, Aarhus University Hospital, Palle Juul-Jensens Boulevard 99, DK-8200 Aarhus N Aarhus, Denmark; 4 Department of Clinical Medicine, Aarhus University, Palle Juul-Jensens Boulevard, DK-8200 Aarhus N Aarhus, Denmark; 5 Department of Radiology, Lillebaelt Hospital Vejle, Vejle, Denmark

**Keywords:** Spondyloarthritis, MRI, Roland-Morris, EuroQol, cohort study, ankylosing spondylitis

## Abstract

**Background:**

The purpose of this study is to determine the incidence of bone marrow oedema (BME) at magnetic resonance imaging (MRI) of the sacroiliac joints (SIJ) in a non- rheumatological population, and to explore whether patient-reported outcome measures are suitable for predicting BME at the SIJ at referral. Furthermore, to investigate the final clinical diagnoses three months after initial SIJ MRI.

**Methods:**

This study was a retrospective cohort study consisting of patients 18–45 years of age that were referred for a SIJ MRI between 1 July 2016 to 30 June 2017 at the Department of Radiology in Lillebaelt Hospital, Denmark. The SIJ MRI radiological reports were evaluated for signs of BME. Principal and secondary diagnoses according to the 10th version of International Classification of Diseases (ICD-10)—three months after the initial MRI—were identified in the electronic patient record system. For a subgroup of patients, patient- reported outcome measures, such as the 23-item Roland Morris Disability Questionnaire, quality of life and pain intensity in the back and leg were included from the local SpineData database.

**Results:**

In total, 333 patients were included, and 187 (56.2%) of those patients received a final diagnosis within three months after the SIJ MRI. BME was detected in 63 (18.9%) patients; 17 (9.1%) patients had both BME at SIJ MRI and were diagnosed with spondyloarthritis (M45/M46). There was no statistically significant difference between patients with and without BME regarding demographics, quality of life, pain descriptions or function.

**Conclusions:**

The incidence of BME in the cohort correlates well to previous studies regarding the incidence of SIJ MRI changes in non-rheumatological populations in Denmark. Patient-reported outcome measures do not seem to contribute to identifying patients with early-phase BME in a non-rheumatological population.

## Background

Magnetic resonance imaging (MRI) of the sacroiliac joints (SIJ) is commonly used to evaluate patients suspected of having early-stage spondyloarthritis (SpA)(1).

Previous studies have shown that bone marrow oedema (BME) at the SIJ is not exclusively present in patients with SpA, but it is also detected in patients with unspecified chronic low back pain (2), in healthy individuals and in women with postpartum pelvic pain (3). In light of the above, there is a risk of misclassification, leading to an overestimation of patients with SpA (4), especially if the MRI findings are over-emphasized compared to other classification criteria for SpA according to The Assessment of SpondyloArthritis international Society (ASAS)(1). Currently, no single biomarker is available to discern between early-stage SpA and low back pain from other causes. The human leukocyte antigen (HLA-B27) lacks specificity as a diagnostic biomarker, and the C-reactive protein (CRP) is only elevated in up to 40% of patients with active SpA (5). Furthermore, no single clinical test or medical history is able to differentiate early SpA from other musculoskeletal conditions in lower back and pelvis, since both degenerative and inflammatory conditions can present with similar symptoms, such as early morning stiffness and relief of pain when using non-steroidal anti-inflammatory drugs (NSAIDs). The treatment possibilities for non-radiographic (nr) SpA and ankylosing spondylitis (AS) have improved tremendously over the last decade with the implementation of biological agents, such as TNF-α inhibitors and interleukin blockers. With new treatment options available, it is important to be able to distinguish patients with SpA in an early phase from patients with low back and pelvic pain due to other causes to ensure the right patients are treated and to reduce overtreatment and extensive health care costs (4). This study was performed to determine the incidence of BME at MRI of the SIJ in a non-rheumatological population in Lillebaelt Hospital, Denmark. Furthermore, we wanted to investigate the clinical conclusion defined by the diagnoses, which were based on the 10th version of the International Classification of Diseases (ICD-10) (6), registered three months after the initial SIJ MRI. Finally, we aimed to explore whether the use of patient reported outcome measures (PROMs), such as EuroQol, the Rolland Morris Disability Questionnaire (RMDQ) and pain descriptions are suitable for predicting BME at the SIJ at baseline and thereby increasing the knowledge about patients where SIJ MRI will be advantageous.

## Methods

This study was a retrospective cohort study consisting of all patients, 18 to 45 years of age, referred for a SIJ MRI between 1 July 2016 to 30 June 2017 (both dates included) at the Departments of Radiology in Lillebaelt Hospital (Middelfart, Kolding, Vejle and Fredericia). The patients could be referred by any hospital department or private practice.

Health Care Service Classification System (SKS) code UXME50 was used to identify relevant patients with a SIJ MRI in the patient record system, Cosmic.

Using the unique Danish CPR-number and date of MRI, the radiological description of SIJ MRI was extracted from the local picture archiving and communication system (PACS) and evaluated regarding information about signs of BME and/or degenerative changes.

In Denmark, the indications for performing SIJ MRI are clinical suspicion of inflammatory disease such as spondyloarthritis, and in rare cases clarification of infection at the SIJ (7).

Only the radiological description of the SIJ was considered when evaluating the MRI examination. No further evaluation or validation of the MRI sequences was made in this study. As a standard procedure, the radiologists and specialised chiropractors in Middelfart and Vejle describe MRI changes in the SIJ according to ASAS recommendations (1). Counter-signature was performed in each case by an experienced radiologic consultant or specialised chiropractor before the final MRI description was released.

MRI technique and sequences: MRI of the entire spine was performed with a 1.5 T Philips Achieva (Best, The Netherlands). MRI System encompassing sagittal T1-weighted and STIR sequences; coronal oblique T1-weighted and T1-fat suppressed (SPIR), and axial oblique STIR sequences were used to evaluate the sacroiliac joints as suggested by the ESSR Arthritis Subcommitee (8).

It is not clear whether all of the SIJ MRI reports from the Department of Radiology, Kolding are described according to ASAS recommendations since tele-radiological assistance from abroad has been used in some cases. For that reason, the MRI findings were divided into two main categories depending on the assessment from the radiologists: 1) BME fulfilling the ASAS criteria for sacroiliitis or explicit radiological description of SIJ changes based on evident inflammation; 2) minor changes not fulfilling criteria for sacroiliitis or no signs of BME (Non-BME).

To identify incident patients, those patients who had a previous MRI examination were excluded. For each patient, information regarding the date of MRI, the radiological department performing the MRI, previous MRI and data of BME and/or degenerative changes in SIJ were manually extracted by one of the authors (AN) and entered into the online-based questionnaire, Survey-Xact (9). From the journal system, Cosmic information regarding the referring medical department, date of first contact and principal and secondary diagnoses—according to the ICD-10—three months after the initial MRI exam were extracted.

Only patients with a principal or secondary code of AS (M.45) or SpA unspecified (M.46) were defined as having spondyloarthritis in this study. All patients with a spondyloarthritis diagnose + another SpA-related diagnoses such as enteropathic arthritis, psoriatic arthropathy, e.g. were defined as a SpA diagnose. Patients without a secondary AS/SpA diagnose were not defined as having spondyloarthritis.

**Fig. 1:**
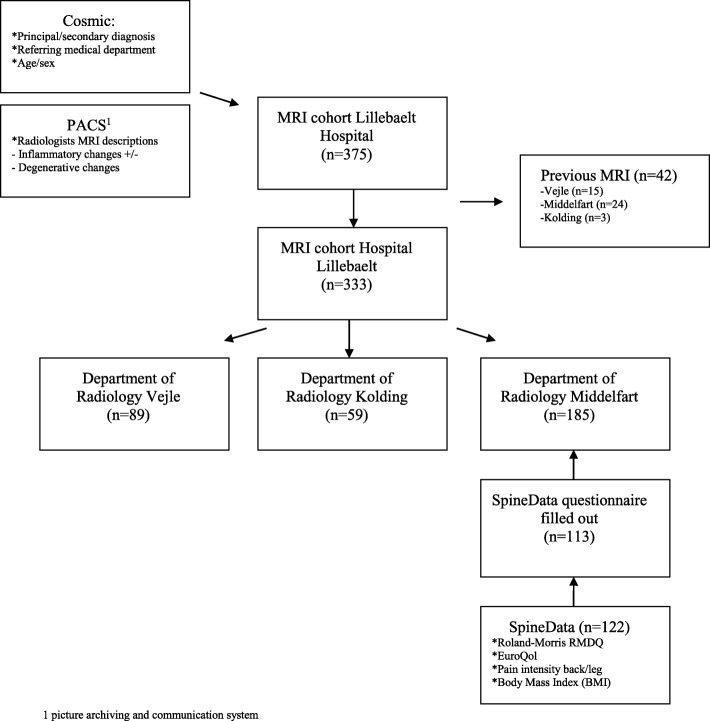
Dataflow and MRI cohort at Lillebaelt Hospital from 1 July 2016 to 30 June 2017. 1 picture archiving and communication system

Patients referred for an MRI from the Department of Medicine, Spine Centre of Southern Denmark, Middelfart had—as a part of their clinical investigation—filled out multiple questionnaires, including the 23-item Roland Morris Disability Questionnaire (RMDQ), quality of life (EQ-5D) and back and leg pain intensity in the SpineData registry, which is an online system used by the patients before initial clinical contact (10) (Fig. 1).

RMDQ has been widely used as a measure of functional limitation due to low back pain with or without leg pain (a higher score indicates a higher level of disability) (11). The 23-item RMDQ has been validated in Danish (12), and a Danish study has shown a higher RMDQ score in patients with MRI-verified inflammatory changes in the SIJ compared to patients with degenerative changes only (13).

In 1990, the EuroQol group developed a health-related quality of life questionnaire (EQ-5D) consisting of five dimensions (mobility, self-care, main activities, social relationships, pain and mood) and an EQ visual analogue scale (score 0–100) to be used for describing a non-disease specific health status (14). Studies on patients with SpA have shown a negative correlation between EuroQol and SpA disease activity scores, such as BASDAI and ASDAS (15–16).

From the collected information in SpineData, analyses were made to explore whether there was a significant difference between patients with and without BME—as determined by MRI—regarding functional limitation, quality of life and back and leg pain intensity, respectively, and demographic data, such as sex, age and BMI.

In case of more than one registry in the SpineData database on the same patient within the inclusion period, the information closest to the date the MRI was performed was used.

### Statistical analysis

STATA software, version 15 was used to perform data analysis. Descriptive data were reported as the mean and standard derivation (SD). One-way analysis of variance (ANOVA) and in between-group comparisons for continuous and categorical demographic variables were performed with the independent sample t-test and Pearson Chi-square test, respectively. The Mann-Whitney U test was used as a nonparametric test, and multiple logistic regression analysis was used to test independent variables as prognostic factors for BME detected by MRI, resulting in odds-ratios. A *p*-value < 0.05 was considered statistically significant.

## Results

A total of 375 patients 18 to 45 years of age were referred for SIJ MRI from 1 July 2016 to 30 June 2017 at Lillebaelt Hospital; 42 (11.2%) of the patients had a previous MRI examination and were excluded. In total, 333 patients were included, and of those, 122 patients had been evaluated clinically at the Spine Centre of Southern Denmark, and 113 patients filled out the SpineData questionnaire (Fig. 1).

A total of 63 (18.9%) patients fulfilled the criteria of BME, as detected by SIJ MRI, compatible with a SpA diagnosis (Table 1). The Department of Radiology, Vejle, had the highest percentage of patients with BME at the MRI exam (27%), but the percentage was not significantly different from the incidence of BME in Kolding and Middelfart.

**Table 1:** BME and degenerative changes at SIJ MRI in the three departments of radiology at Lillebaelt Hospital

	Total	Vejle	Middelfart	Kolding	*p*-value
Age, years (SD)	33.1 (7.89)	32.4 (7.37)	33.7 (7.99)	32.1 (8.22)	0.60^1^
Sex, *n* (%)					0.21^2^
Male	106 (31.8)	35 (39.3)	54 (29.2)	17 (28.8)	
Female	227 (68.2)	54 (60.7)	131 (70.8)	42 (71.2)	
MRI, *n* (%)					
BME	63 (18.9)	24 (27.0)	30 (16.2)	9 (15.3)	0.08^2^
Non-BME	270 (81.1)	65 (73.0)	155 (83.8)	50 (84.7)	
Degenerative. changes	17 (5.1)	4 (4.5)	12 (6.5)	1 (1.7)	0.33^2^

^1^One-way ANOVA, ^2^Pearsons Chi2 test

In 60 (18.1%) patients, the radiologists reported minor signs of BME detected by the SIJ MRI exam that did not fulfil the ASAS criteria for SpA; 17 (5.1%) patients had degenerative SIJ changes based on MRI. Overall, 193 (58.0%) cases had no changes in the SIJ.

The mean age of the population was 33.1 years of age (standard deviation (SD): 7.89), with no significant differences between the BME (32.7, SD: 7.59) and non-BME (33.3, SD: 7.95) groups. The groups primarily consisted of women in both the BME (63.5%) and non-BME (69.3%) groups, with no significant differences between the groups.

In total, 187 (56.2%) patients were registered with a diagnosis within 90 days after the initial SIJ MRI scan. Table 2 presents the identified MRI changes and AS/SpA diagnosis among the different diagnostic departments in Lillebaelt Hospital.

**Table 2:** BME changes detected by SIJ MRI and the use of diagnoses

	Vejle (*n* = 49)	Middelfart (*n* = 132)	Other^1^ (*n* = 6)	Total (*n* = 187)
SIJ MRI				
BME, *n* (%)	15 (30.6)	21 (16.0)	2 (33.3)	38 (20.3)
Non-BME, *n* (%)	34 (69.4)	111 (84.0)	4 (66.6)	149 (79.7)
Diagnoses, *n* (%)				
AS^a^ (M.45.9)	3 (6.1)	2 (1.5)	–	5 (2.7)
SpA^b^ (M.46)	9 (18.4)	9 (6.8)	–	18 (9.6)

^1^Department of Rheumatology in Kolding and Fredericia. ^a^Ankylosing spondylitis, ^b^Spondyloarthritis.

A total of 38 (20.3%) patients had BME at SIJ MRI; 17 (9.1%) patients had BME at the SIJ MRI and were diagnosed with AS/SpA (M45/M46); 21 patients had BME, but no AS/SpA diagnosis, 6 patients received a SpA diagnosis without BME at SIJ MRI. In total, 23 (12.3%) patients were diagnosed with AS/SpA within 90 days from the initial SIJ MRI.

In the 63 patients with BME identified at SIJ MRI, 36.5% were diagnosed with AS/SpA within three months. The most frequent diagnosis among the patients having BME at SIJ MRI, but no AS/SpA diagnosis was low back pain (M.54.5) and dorsalgia unspecified (M.54.9), with six cases for each diagnosis.

Fifteen patients were referred for SIJ MRI by their general practitioner (GP); eight of them (53.3%) had BME at SIJ MRI. The mean age was 31.9 years (SD: 6.55) by the time of the referral, and the majority of patients were women (60%). All of the patients received a final diagnosis within 90 days from the initial SIJ MRI, and four (26.6%) cases were diagnosed with SpA (M.46), while none were diagnosed with AS (M45). The 15 patients referred for SIJ MRI from their GP were diagnosed at the Spine Centre of Southern Denmark (*n* = 7), Vejle (*n* = 7) and Fredericia (*n* = 1), respectively.

A total of five patients were referred for SIJ MRI from rheumatology private practices; one (20%) case had BME at SIJ MRI. The mean age was 36.6 years (SD: 4.39), and the majority of patients were male (80%). Four cases were diagnosed within 90 days, and none of them were AS or SpA.

### Inflammatory diagnoses

**Fig. 2:**
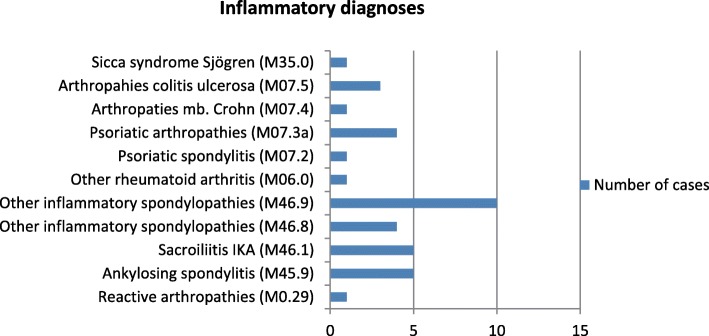
Frequency of AS/SpA and other inflammatory associated diagnoses

Fig. 2 presents the frequency of AS/SpA and other arthritis associated diagnoses among the entire population of patients who received a diagnosis within 90 days after SIJ MRI.

In total, 10 patients were diagnosed as having inflammatory bowel disease, psoriatic arthropathy or reactive arthritis. All of the patients diagnosed with Mb. Crohn (M0.74) and colitis ulcerosa (M0.75) had a principal diagnosis of SpA (M.46) registered within the first visit after SIJ MRI.

None of the four patients with psoriatic arthropathy (M0.73) were diagnosed with SpA. BME was diagnosed by SIJ MRI in one patient with psoriatic spondylitis (M0.72) and psoriatic arthropathies (M0.73), respectively.

In total, 113 of 122 (93%) patients who were referred for SIJ MRI at the Radiology Department Middelfart, and examined at SpineCentre of Southern Denmark, Middelfart filled out the PROMs in the SpineData database. The mean age was 33.3 years (SD: 7.89), and 78 (69%) patients were women. The mean BMI was 26.7, with a trend toward a higher BMI in the group with BME (non-significant).

A total of 16 patients had BME according to SIJ MRI (males *n* = 3, females *n* = 13), and 7 patients with BME had an AS/SpA diagnosis. Of the 97 patients without BME at SIJ MRI, 2 patients were diagnosed with SpA (M.46), and none were diagnosed as AS (M.45). Degenerative changes were detected with MRI in eight patients (7%).

Of the 113 patients who filled out the RMDQ, 104 (92%) completed the questionnaire without missing data. In four cases, there were missing data from 2, 3 and 7 questions, respectively, five patients did not answer any of the RMDQ questions, all of them were non-BME cases based on the SIJ MRI. The mean RMDQ proportional score was 61.3 with a range of 0–100.

Of the 113 patients, 106 (94%) filled out the EuroQol questionnaire, 5 patients did not answer any of the five EuroQol questions; none of them had BME at SIJ MRI. The mean EuroQol thermometer was 50.4, with a range of 0–100.

Overall, there were no significant differences between the BME and non-BME populations in SpineData regarding age, sex, BMI, the mean RMDQ or the EuroQol health thermometer (Table 3). Only two patients registred in SpineData were diagnosed with AS. The mean RMDQ for those patients were higher than the average for the group with BME (mean RMDQ: 74).

**Table 3:** SpineData Demographics in the BME and non-BME patients

	BME (*n* = 16)	Non-BME (*n* = 97)	*p*-value
Age, years (SD)	35 (7.17)	33 (8.00)	0.35^a^
Sex, *n* (%)			0.25^b^
Male	3 (18.6)	32 (33)	
Female	13 (81.4)	65 (67)	
BMI, Mean (SD)	27.9 (7.35)	26.5 (4.97)	0.36^a^
Disease activity			
RMDQ (0–100), Mean (SD)	68.6 (21.6)	60.0 (62.7)	0.16^a^
Low back pain intensity, (0–10): Mean (SD)	5.75 (2.54)	5.47 (2.37)	0.55^c^
Leg pain intensity (0–10), Mean (SD)	4.0 (2.78)	3.70 (2.95)	0.71^c^
EuroQol health thermometer (0–100), Mean (SD)	52.8 (24.0)	49.9 (21.0)	0.63^a^

^a^t-test, ^b^Pearson Chi2 test, ^c^Mann-Whitney U test

The logistic regression on demographics (age, sex, BMI), pain (present leg and back pain) and function (Rolland Morris proportional score and EuroQol sum score) were tested (Table 4), and there were no significant differences between the BME and non-BME groups.

**Table 4:** Grouped logistic regression analysis of pain, function and demographics between groups

Variables	Odds Ratio (95% CI)	*p*-value
Demographics		
Age	1.01 (0.94–1.09)	0.78
Sex	0.54 (0.14–2.11)	0.37
BMI	1.04 (0.94–1.15)	0.41
Pain		
Low back pain (present)	1.03 (0.77–1.37)	0.87
Leg pain (present)	1.02 (0.81–1.29)	0.85
Function		
RMDQ proportional score	1.02 (0.99–1.05)	0.20
EuroQoL sum score	1.02 (0.99–1.06)	0.17

## Discussion

The primary purpose of this study was to identify the incident cases of BME in patients referred to an MRI of the SIJ at Lillebaelt Hospital, Denmark. BME was identified in 18.9% of the SIJ MRI examinations (*n* = 63), which correlates well to previous studies regarding the incidence of MRI changes in non-rheumatological populations in Denmark (17).

In the present study, both the BME and non-BME groups primarily consisted of women. An explanation for the relatively high number of women in the cohort could be that patients with postpartum pelvic pain/pelvic dysfunction often have symptoms that mimic those of inflammatory back pain due to early SpA (18), which might lead to a higher percentage of women being referred for SIJ MRI.

The majority of the patients (56%) were examined at the Department of Radiology, Middelfart and were examined and diagnosed at the Spine Centre of Southern Denmark. The Department of Radiology, Vejle, had the highest percentage of patients with BME detected by SIJ MRI. It is plausible to believe that patients referred for SIJ MRI from a rheumatological expert/department have a higher frequency of BME than patients referred from other departments. The Department of Radiology, Kolding, had the lowest number of cases of BME at the SIJ MRI, and none of them were diagnosed with AS/SpA. A reason for the differences in the incidence of BME changes between the departments might be that the rheumatological department in Vejle is the largest in Lillebaelt Hospital, and therefore it is reasonable to refer patients highly suspicious for SpA for SIJ MRI at the same location where the patients would be followed in case of a SpA/AS diagnosis.

Only a few patients were referred for SIJ MRI from a rheumatological private practice, which may reflect the small number of private practising rheumatologists in the Region of Southern Denmark (19). Another explanation might be that the majority of patients with low back pain in Southern Denmark are initially referred to the Spine Centre of Southern Denmark and Vejle Hospital rather than to private practising rheumatologists. About one-third of the patients with BME, diagnosed by SIJ MRI, received a SpA diagnosis within three months. The vast majority of patients without AS/SpA were diagnosed with unspecified low back pain, joint pain and other musculoskeletal disorders. In these cases, the clinical presentation, patient history and MRI changes must have been evaluated and concluded by the clinician to be inconsistent with SpA—suggesting that clinicians working with patients with low back pain are aware of the fact that minor changes observed in SIJ MRI can be of a biomechanical origin as well.

A small number of patients were diagnosed with SpA, without BME based on MRI. Theoretically, these patients could have fulfilled the non-imaging arm of ASAS SpA classification criteria, but there is also a risk of misclassification of these patients. Only five patients were diagnosed with AS, which is not surprising since this diagnosis requires radiographical changes according to the Modified New York Criteria, which usually takes years to develop (20). Whether these patients had radiography examinations before the diagnosis is unknown.

There were no significant differences in demographics or pain characteristics between the BME and non-BME groups in SpineData. There was a non-significant trend toward a higher RMDQ score in the BME group, but the RMDQ score does not seem to be a valid variable to discriminate between BME and non-BME patients in this study. The major reason for this could be the small number of cases with BME detected by MRI in the SpineData group (*n* = 16). Another reason for the non-significant difference between the groups could be that only two AS patients were registered in SpineData. It is possible that a higher frequency of AS patients would have increased the RMDQ score in the BME group. The results highlight the fact that it is challenging to differentiate low back and pelvic pain on a biomechanical basis from inflammatory pain in the early stage of disease. In the present study, information regarding the duration and debut of symptoms prior to SIJ MRI was not evaluated.

### Strengths and limitations

A limitation of this study is the use of radiological reports to decide whether or not the patients had BME at SIJ MRI. It is possible that the radiologists and specialised chiropractors emphasised or understated the degree of changes in the MRI report depending on the clinical question asked, even though the radiologists in Vejle and Middelfart refer to the guidelines from ASAS when describing changes observed in MRI examinations. Furthermore, it is uncertain whether all of the SIJ MRI examinations performed at Kolding were described in accordance with the recommendations of ASAS. However, over 80% of the total SIJ MRI examinations were described by the same group of radiologists and specialised chiropractors at both Vejle and Middelfart. The small number of AS patients and patients with BME at the time of MRI in the SpineData cohort is another limitation. It is possible that some PROMs like the RMDQ would have shown a statistical difference between the BME and non-BME groups in a larger population. A strength of this study is that all patients who underwent SIJ MRI within a twelve months period at Lillebaelt Hospital are represented in the present study, and this contributes to knowledge regarding the incidence of SIJ MRI in a non-rheumatological population.

## Conclusions

In conclusion, the incidence of BME at SIJ MRI in the present study correlates well with previous studies regarding the incidence of MRI changes in non-rheumatological populations in Denmark. In light of the above results, it seems that demographics and pain characteristics are not of significant interest when categorising patients into either the BME or non-BME group. It is possible that RMDQ can be of use in a future study, but demographics like age, sex, BMI and pain descriptions do not seem to add valuable information regarding the distinction between patients with and without BME in an early stage. To avoid potential challenges when interpreting BME changes, it would be preferable if all MRI examinations were reviewed according to the same criteria and by the same radiologist/ specialised chiropractor.

## Data Availability

The datasets used and/or analysed during the current study are available from the corresponding author upon reasonable request.

## Abbreviations

AS: Ankylosing spondylitis
ASAS: Assessment of SpondyloArthritis International Society
ASDAS: Ankylosing Spondylitis Disease Activity Score
BASDAI: Bath Ankylosing Spondylitis Disease Activity Index
BME: Bone marrow oedema
CRP: C-reactive protein
ICD-10: 10th version of International Classification of Diseases
MRI: Magnetic resonance imaging
NSAID: Non-Steroidal Anti-Inflammatory Drugs
PACS: Picture archiving and communication system
PROM: Patient-reported outcome measures
RMDQ: Rolland Morris Disability Questionnaire
SIJ: Sacroiliac joints
SKS: Health care Service Classification System
SpA: Spondyloarthritis
